# The Influence of Oviductal and Uterine Fluid Supplementation on the In Vitro Development and Quality of Cloned Sheep Embryos

**DOI:** 10.3390/ani14192894

**Published:** 2024-10-08

**Authors:** José Roberto Vazquez-Avendaño, César Cortez-Romero, Demetrio Alonso Ambríz-García, José Luis Rodríguez-Suástegui, José Ernesto Hernández-Pichardo, María del Carmen Navarro-Maldonado

**Affiliations:** 1 Doctorado en Ciencias Biológicas y de la Salud, Universidad Autónoma Metropolitana, México City 3855, México; robertmizer@gmail.com; 2 Department of Biology of Reproduction, Division of Biological and Health Sciences, Universidad Autónoma Metropolitana Unidad Iztapalapa, México City 09310, México; deme@xanum.uam.mx; 3 Program in Genetic Resources and Productivity-Livestock, Campus Montecillo, Colegio de Postgraduados, Texcoco de Mora 56264, México; ccortez@colpos.mx; 4 Program in Innovation in Natural Resources Management, Campus San Luis Potosí, Colegio de Postgraduados, Salinas de Hidalgo 78620, México; 5 Department of Agriculture and Animal Production, Division of Biological and Health Sciences, Universidad Autónoma Metropolitana Unidad Xochimilco, México City 04960, México; embrioninvitro@hotmail.com (J.L.R.-S.); ehernan@correo.xoc.uam.mx (J.E.H.-P.)

**Keywords:** nuclear transfer, embryonic development, oviductal fluid, uterine fluid, reactive oxygen species, glutathione

## Abstract

**Simple Summary:**

The in vitro culture of mammalian embryos lacks different embryotrophic factors that are present in the female reproductive tract and are essential for correcting embryonic development. It has been reported that supplementation with oviductal fluid (OF) and uterine fluid (UF) during the in vitro culture of mammalian embryos produced by IVF has a positive effect on their development and quality. We investigated the effect of OF–UF supplementation on the balance of reactive oxygen species (ROS), glutathione (GSH), and the development of cloned and parthenogenetic *Ovis aries* embryos. However, at concentrations of 1 and 2% OF–UF, the blastocyst rate decreased in both groups of embryos. At a concentration of 1% OF–UF, both intracellular ROS and GSH decreased in the blastocysts of parthenogenetic embryos but not in cloned embryos. In cloned sheep embryos, OF–UF had no impact on the embryonic development or redox balance.

**Abstract:**

Somatic cell nuclear transfer (SCNT) has great potential for the replication of high-commercial-value animals, threatened wild species for conservation purposes, and transgenic animals for biomedical purposes. However, SCNT has a low success rate due to intrinsic factors of the technique itself, which leads to low rates of embryonic development and epigenetic alterations in cloned embryos. The objective of this study was to evaluate the effect of OF–UF on the intracellular concentrations of ROS and GSH and the development of cloned and parthenogenetic *Ovis aries* embryos. The results do not show a beneficial effect on the development of parthenogenetic and cloned embryos at concentrations of 0.5% OF–UF. Furthermore, at 1% OF–UF, an adverse effect was observed in cloned embryos at the blastocyst stage and 2% OF and UF in parthenogenetic embryos during the first divisions. Decreases in ROS and GSH levels were observed in the parthenogenetic blastocysts treated with 1% OF–UF, but not in the clones, in which a higher concentration of GSH and a similar concentration of ROS were observed. No effect of OF–UF was observed on embryonic development and redox balance in sheep embryos cloned via handmade cloning.

## 1. Introduction

According to data from the International Embryo Technology Association (IETS), the in vitro production of sheep embryos increased more than 340% from 2021 to 2022 [1], being one of four productive species that have demonstrated growth and efficiency in the use of assisted reproduction techniques (ARTs); the other three species are cattle, goats, and horses. Among ARTs, SCNT allows for the multiplication of individuals with genetic characteristics, for example, animals of zootechnical interest and high commercial value [2], threatened wild species for conservation purposes [3,4], companion animals [5], and transgenic animals for biomedical purposes [6].

However, this biotechnology still does not have a practical application like other ARTs, due to the small number of cloned embryos that result in healthy offspring. In sheep, efficiencies of 5.3 to 42% of cloned embryos reaching the blastocyst stage have been reported, while the efficiency in newborn cloned lambs per blastocyst transferred is 5.7 to 15% [7]. This is because of limitations inherent to the technique, such as trauma due to manipulation of the cells used, inadequate capacity of the recipient oocyte to reprogram the somatic cell nucleus, somatic cell resistance to reprogramming, or abnormalities induced by the in vitro culture [8].

This leads to epigenetic alterations in embryos produced by SCNT, such as elevated levels of DNA methylation in cloned embryos, compared to those produced by IVF or in vivo [9]. This has been observed in sheep, particularly in the trophectoderm cells of the blastocyst [10], which give rise to placentas with a reduced number or abnormalities in the placentomes and with histopathological alterations [11,12,13]. Newborn cloned lambs have also been described as having a high incidence of abnormal offspring syndrome (AOS) due to the variety of reported phenotypes [14]. This is related to a deregulation of different imprinted genes [15,16,17].

Although the media used have supported the in vitro development of embryos, there is increasing evidence of abnormalities present in embryos at the genetic and epigenetic levels [18,19,20,21] as these media lack proteins present in the oviduct and uterus [22], whose secretions are mainly influenced by the hormones secreted throughout the estrous cycle.

Different studies have evaluated the effects of oviductal fluid (OF) and uterine fluid (UF) on embryos produced using IVF in cattle [23,24,25,26] and pigs [27]. These studies have reported no benefits from supplementation with OF and UF on the blastocyst rate; however, they have found an improvement in embryo quality [24,27].

Sequential supplementation of OF and UF recovered from the late follicular phase and early luteal phase, respectively, during the in vitro culture of porcine embryos did not increase the total blastocyst rate but did increase the rate of hatching (0 vs. 15.4%, *p* < 0.05) and hatched blastocysts (0 vs. 5.1%, *p* < 0.05) [27].

On the other hand, a positive effect of OF and UF was observed on the cryotolerant capacity of vitrified embryos. Bovine embryos treated with 0.625 and 1.25% OF and vitrified presented a higher percentage of re-expansion (53.6 and 57.7%) vs. 2.5% OF (36.1%, *p* < 0.05) [24]. The sequential addition of OF and UF has a positive long-term effect on embryonic survival after heating vitrified embryos. A total of 62.8% of blastocysts cultured in 1.25% OF and UF remained viable in culture at 48 and 72 h post-vitrification, while blastocysts cultured in FCS or BSA gradually decreased their viability as the culture time increased. In the group treated with FCS, the viability was 41.1 to 31.6% (*p* > 0.05), while that treated with BSA had 73.3 to 64.8% viability (*p* > 0.05) [25].

Lopera-Vasquez et al. [24] reported supplementation with 1.25 and 0.625% OF during the culture of bovine embryos produced using IVF. This significantly increased the number of cells present in blastocysts (165.1 and 156.2, respectively, *p* > 0.05), when compared to embryos cultured with and without SFB (143.1 and 127.7, respectively, *p* > 0.05). In porcine blastocysts produced using IVF treated with OF and UF, the number of cells was similar to that of blastocysts obtained in vivo (81.8 vs. 87, respectively) [27]. Hamdi et al. [25] observed that embryos cultured with OF and UF presented a significant decrease (*p* < 0.001) in the presence of reactive oxygen species (ROS) compared to embryos cultured with FCS and BSA. This was due to a decreased expression of genes related to oxidative stress (CLIC1) and the enzyme glutathione peroxidase (GPX1). Embryos produced using SCNT typically have higher levels of ROS than embryos produced using IVF [28,29,30], due to the excessive manipulation that cells receive during enucleation, fusion, and activation [31]. The imbalance between the generation of ROS and the antioxidant system of embryos results in oxidative stress. This has a negative impact during embryonic development, which can extend to fetal development [32].

Given that OF and UF contain elements that promote development and improve the quality of embryos produced in vitro, then the supplementation of these fluids during the in vitro culture of cloned *Ovis aries* embryos may improve embryonic quality. Therefore, the aim of this study was to evaluate the effects of OF and UF on the balance of ROS and GSH and the development of cloned and parthenogenetic *O. aries* embryos.

## 2. Materials and Methods

All reagents used were purchased from Sigma-Aldrich. The incubation conditions for cell cultures, in vitro oocyte maturation, and in vitro embryonic development were 38.5 °C, 5% CO_2_, and saturation humidity.

### 2.1. Obtaining Reproductive Fluids (OF and UF)

Reproductive tracts (RTs) of adult Creole type sheep (*Ovis aries*) were collected from a local slaughterhouse and transported to the laboratory at 8 °C in disinfected plastic bags. Upon arrival, within no more than two hours, the RTs were washed in sterile Dulbecco’s Phosphate-Buffered Saline (DPBS) supplemented with 1% antibiotic–antimycotic (streptomycin 10,000 µg mL^−1^, amphotericin B 25 µg mL^−1^_,_ and 10,000 IU of penicillin, In Vitro S.A. from C.V. Ciudad de México, México) at 4 °C. Subsequently, the RTs were classified based on the ovarian structures present (to determine the estrous cycle phase), with regular size and shape, and without cysts, according to the methodology described and adapted for sheep by Carrasco et al. [33]. The luteal phase was divided into early and middle phases. The early phase was determined by the presence of corpora hemorrhagicum (CH), while the middle phase was determined by the presence of developing corpora lutea (CL). The OF was recovered directly, without flushing it with any solution, from the oviduct ipsilateral to the ovary with the presence of CH (early luteal phase), while UF was retrieved from the uterine horn ipsilateral to the ovary with the presence of developing CL (middle luteal phase). Once the RTs were classified, the suspensory ligaments were dissected, and the uterine–tubal junction was sectioned to separate the oviduct from the uterine horn. The oviducts and uterine horns were maintained in DPBS at 4 °C to prevent protein degradation.

The OF was extracted using mechanical compression with a slide by placing the oviduct on a flat surface and pressing it with a slide from the utero-tubal junction toward the ampulla. A fire-polished Pasteur pipette was then introduced in the ampulla to recover the OF, which was deposited in a 300 µL tube. The sample was centrifuged at 2000× *g* for 5 min at 4 °C, and the supernatant was recovered and centrifuged at 7000× *g* for 10 min at 4 °C. Finally, the supernatant was recovered and stored at −80 °C until use. To obtain the UF, the RTs were placed vertically so that the fluid descended by gravity to the uterine–tubal junction. Immediately afterward, a fire-polished Pasteur pipette was introduced inside this junction, and the UF was collected and placed in a 300 µL tube to be processed and stored in the same way as the OF.

### 2.2. Obtaining and Culturing Skin Fibroblasts for Use as Karyoplasts

Ear skin fibroblasts were obtained according to the method of Navarro-Maldonado et al. [34]. First, an ear skin sample was obtained from an adult sheep (*O. aries*), and fibroblasts were obtained using mechanical explant sowing and enzymatic digestion. Then, the cells were cultured in DMEM (Dulbecco’s Modified Eagle’s Medium, In Vitro S.A. from C.V. Ciudad de México, México) supplemented with 2.3% antibiotic–antimycotic, 10% newborn calf serum (NCS), and 13.6 mg mL^−1^ NaHCO_3_. The fibroblasts were taken to the 4th cell passage and cryopreserved at −196 °C. A week before SCNT, the cells were re-seeded and left at confluence (>90%) for at least three days so that they could reach the 5th passage and synchronize their cell cycle (G0/G1) by contact inhibition, before they were used as karyoplasts (nucleus donors).

### 2.3. In Vitro Oocyte Maturation (IVM)

Cumulus–oocyte complexes (COCs) were obtained from the ovaries of slaughterhouse sheep, with follicle aspiration (2 to 8 mm in diameter) using 10 mL syringes and 20 G gauge needles. The aspiration medium was tissue culture medium (TCM-199) with Hepes (In Vitro S. A. from C. V. Ciudad de México, México), supplemented with 100 IU mL^−1^ of heparin. The collected follicular fluid was passed through a cell filter (100 μm, Biologix, Shandong, China) to recover the COCs, which were selected based on their cytoplasmic morphology and number of granulosa cell layers [35]. The selected COCs were cultured in 4-cell dishes (Nunc™) filled with 0.5 mL of in vitro maturation medium (IVM, based on TCM-199, In Vitro S. A. from C. V. Ciudad de México, México) supplemented with 1.675 × 10^−4^ mg.mL^−1^ of cysteine, 0.001 mg.mL^−1^ of polyvinyl alcohol, 5.496 × 10^−4^ mg.mL^−1^ of D-glucose, 1 × 10^−4^ mg.mL^−1^ of sodium pyruvate, 10% newborn calf serum (NCS), 5 IU mL^−1^ of equine chorionic gonadotropin (eCG, Gonaforte Parfarm S.A., Ciudad de México, México), 0.1 IU mL^−1^ of recombinant follicle-stimulating hormone (FSH, Gonal-F Merck, Darmstadt, Germany), 10 ng mL^−1^ of epidermal growth factor (EGF), and 0.65% antibiotic–antimycotic. The COCs were incubated for 21 h at 38.5 °C, 5% CO_2_, and saturation humidity.

### 2.4. Somatic Cell Nuclear Transfer (SCNT) through Handmade Cloning (HMC)

Once IVM was complete, SCNT was performed through handmade cloning (HMC). The COC expanded cumulus cells were disintegrated by incubating them for 8 min in 0.5 mg mL^−1^ of hyaluronidase, followed by gentle pipetting. The oocytes in MII (showing the presence of the first polar body) were selected and incubated in 0.5 µg mL^−1^ of demecolcine in IVM medium for 1 h to induce the cytoplasmic extrusion of the genetic material (first polar body and metaphase plate) [36,37].

#### 2.4.1. Cytoplast Preparation

Subsequently, the zona pellucida (ZP) was removed from the oocytes in 2 mg mL^−1^ of pronase in 30 µL drops of TCM-199 medium with 4-(2-hydroxyethyl) piperazine-1-ethane-sulfonic acid (HEPES). Zona pellucida-free oocytes were manually enucleated with a microblade (Professional Embryo Transfer Supply, Inc., Canton, TX, USA), in 30 µL drops of TCM-199 with HEPES and 10% NCS (T10). For enucleation, the oocytes were placed, orienting the extruded portion of their cytoplasm (the one that contained the genetic material) at the north pole, and then the microblade was passed, manually sectioning that portion of the cytoplasm. The enucleated oocytes (cytoplasts) were placed in 30 µL drops of TCM-199 with HEPES and 20% NCS (T20) for reconstitution, that is, until they returned to their spherical shape, as a sign of viability [36,38].

#### 2.4.2. Triplets’ Formation and Fusion

Groups of 5 cytoplasts were immersed in 5 mg mL^−1^ of phytohemagglutinin in TCM-199 medium with HEPES and 2% NCS (T2) for 3 sec. Pairs of cells were formed (karyoplast–cytoplast), dropping each cytoplast on a fibroblast (karyoplast). Then, triplets (cytoplast–karyoplast–cytoplast) were formed and equilibrated in fusion medium (0.3 M of D-mannitol and 1 mg mL^−1^ of polyvinyl alcohol), aligned on the positive electrode of the fusion chamber (BTX microslide, 0.5 mm aperture, model 450, Holliston, MA, USA). The triplets were fused by a single pulse of 1.0 kV cm^−1^ of direct current (DC) for 9 µs, and then placed in drops of T20 for reconstitution to form a single cell. Meanwhile, the fusion process was repeated with the remaining cytoplasts. Once the triplets were reconstituted in a single cell, they were incubated in in vitro development medium (IVD) for 1.5 h to promote the nuclear reprogramming of the donor cell [38].

#### 2.4.3. Activation of Cloned Embryos

Cloned embryos were activated in 8 µg mL^−1^ of Ca^2+^ ionophore A23187 in TCM-199 with HEPES and 2% NCS (T2) for 5 min, and then in 2 mM 6-DMAP in IVD medium (Cleavage, Cook Medical, Bloomington, IN, USA) for 4 h, under the same incubation conditions.

#### 2.4.4. In Vitro Culture of Cloned Embryos in the WOW System

According to Vajta et al. [39], cloned embryos were cultured in the Well of the Well (WOW) system, which consisted of making microwells with a punch inside a cell of a 4-cell box (Nunc™) and placing a single-cloned embryo in each microwell. They were cultured for 4 days in IVD cleavage medium (Cook Medical, Bloomington, IN, USA) and subsequently in blastocyst medium (Cook Medical, Bloomington, IN, USA) for three more days until they reached the blastocyst stage on day seven.

Embryos from the experimental groups were cultured in IVD supplemented with different concentrations of OF and UF, as follows. First, the embryos were cultured in cleavage medium supplemented with OF (0.5, 1.0, or 2.0%) for 4 days. Then, the embryos were cultured in blastocyst medium supplemented with UF (0.5, 1.0, or 2.0%), and then cultured for 3 more days to complete 7 days of culture. Three experimental groups were established, which consisted of 0.5% OF–UF, 1.0% OF–UF, and 2.0% OF–UF.

### 2.5. In Vitro Production of Parthenogenetic Embryos

After IVM, MII oocytes were selected and activated following the same method described for the cloned embryos. They were cultured in cleavage medium for 4 days and in blastocyst medium for 3 more days, for a total of 7 days. The experimental groups were processed separately and supplemented in the same way as the cloned embryos, with 0.5, 1.0, and 2.0% OF and UF.

### 2.6. Evaluation of ROS and GSH Levels in Parthenogenetic and Cloned Embryos

According to Nadri et al. [30], the ROS and GSH levels were evaluated in *O. aries* parthenogenetic and cloned embryos at the blastocyst stage (7 days IVD), which were incubated with H2DCFDA (2′,7′-dichlorodihydrofluorescein diacetate, Invitrogen, Eugene, OR, USA), to determine the presence of ROS, and with Cell Tracker Blue™ (Thermo Fisher Scientific, Waltham, MA, USA), to determine the presence of GSH. For this, the blastocysts of each group were washed in DPBS with 1 mg mL^−1^ of polyvinyl alcohol and then incubated with 10 mM H2DCFDA and 10 mM Cell Tracker Blue™ at 38.5 °C for 30 min, protected from light. The embryos were fixed with 4% paraformaldehyde, mounted on slides, covered with coverslips, and sealed with nail polish. They were subsequently evaluated in an epifluorescence microscope using 460 nm UV filters to evaluate the ROS and 405 nm UV filters to evaluate the GSH. The taken images were analyzed with the Image J program (version 1.53; Wayne Rasband, National Institute of Health, Bethesda, MD, USA).

### 2.7. Statistical Analysis

The data were expressed as the mean ± standard error. The differences between the means of the different stages of embryo development, as well as the means of the relative fluorescence intensity of ROS and GSH were analyzed using a one-way ANOVA test. To evaluate the possible relationship between the ROS and GSH, the Spearman correlation test was used. The differences were considered significant at *p* < 0.05. For statistical analysis, the GraphPad Prism program (version 9.5., Boston, MA, USA) was used.

## 3. Results

### 3.1. Effects of OF and UF on the In Vitro Development of Parthenogenetic Embryos

No significant differences were observed in the development of sheep parthenogenetic embryos treated with 0.5 and 1.0% OF and UF (*p* > 0.05) when compared to the control group (Table 1). Although it was observed that there was a higher rate of 4-to-16-cell embryos at 2% OF and UF when compared to the control group (100 vs. 31.8%, respectively, *p* < 0.05), these embryos did not continue their development (Table 1). For this reason, this concentration was not considered for cloned *O. aries* embryos.

### 3.2. Effects of OF and UF on the In Vitro Development of Cloned Embryos

No differences were observed in the development of cloned embryos treated with 0.5% OF and UF when compared to the control group (*p* > 0.05, Table 2). An increase in the 4-to-16-cell rate was observed in embryos treated with 1.0% OF and UF when compared to the control group (62.1 vs. 27.9%, respectively, *p* < 0.05, Table 2). However, a decrease in the morula and blastocyst rates was observed vs. the control group (27.9 vs. 47.3 and 7.7 vs. 28.1%, respectively; *p* < 0.05, Table 2).

### 3.3. Effects of OF and UF on the Presence and Levels of ROS and GSH in Parthenogenetic Embryos

**Figure 1:**
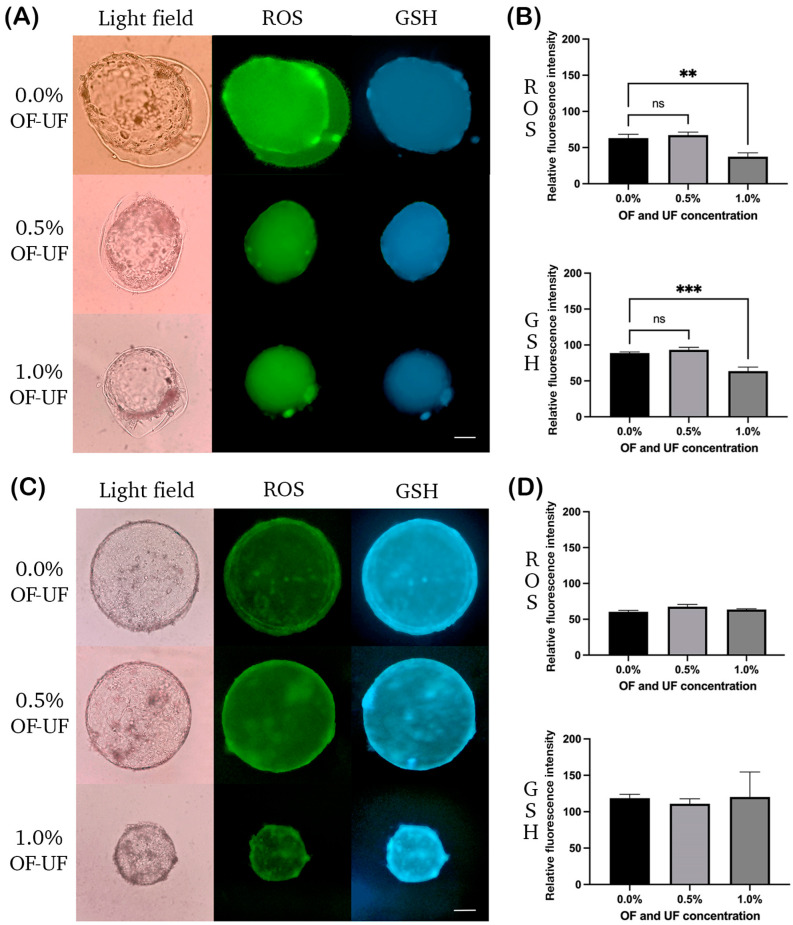
Effects of OF and UF on the presence of ROS and GSH in sheep parthenogenetic and cloned embryos at the blastocyst stage. Panel photographs (light and dark fields) of (**A**) parthenogenetic and (**C**) cloned embryos in the blastocyst stage were observed. The presence of ROS is shown as green fluorescence and GSH as blue fluorescence. Magnification: 100X. Scale bar: 200 μm. Relative fluorescence intensity of ROS and GSH in (**B**) parthenogenetic blastocysts and (**D**) cloned blastocysts analyzed with Image J software. Bars represent the mean ± S.E. *** *p* = 0.0009, ** *p* = 0.0032, no significant difference (ns).

Figure 1A shows parthenogenetic blastocyst stained to determine the ROS and intracellular GSH. No significant differences were observed in the ROS and GSH levels in the parthenogenetic blastocysts treated with 0.5% OF and UF with respect to the control group (Figure 1B).

At 1.0% OF and UF, the ROS and GSH levels decreased significantly when compared to the control group (*p* < 0.05) (Figure 1B). At this same concentration, both variables presented a directly proportional positive correlation (r = 0.8727; *p* < 0.001). That is, as the ROS levels increased, the GSH levels also increased, and vice versa.

At 2.0% OF and UF, there was no development into blastocysts.

### 3.4. Effects of OF and UF on the Presence and Levels of ROS and GSH in Cloned Embryos

Figure 1C shows cloned blastocysts stained to determine the ROS and intracellular GSH. No effect was observed on the levels of ROS and GSH for blastocysts treated with 0.5 and 1% OF and UF, with respect to the control group (Figure 1D).

The levels of ROS and GSH presenting blastocysts in the control group showed a negative, inversely proportional correlation (r = −0.8095; *p* < 0.022). That is, as the GSH levels increased, the ROS levels decreased.

## 4. Discussion

Somatic cell nuclear transfer (SCNT) has great potential in the propagation of animals with genetic and phenotypic characteristics of productive, ecological, or biomedical interest. However, its practical application has been limited due to intrinsic factors of the technique itself [8], which promote alterations at the genetic and epigenetic levels from the beginning of preimplantation embryo development to fetal development. In some cases, and if carried to term, clonal offspring may present AOS [14].

Given the previously described positive effects of OF and UF on the development and quality of embryos produced using IVF [23,24,25,26,27], in this study, their effects on the in vitro development of cloned sheep embryos produced through handmade cloning (HMC) were investigated, something that has not been reported before, making this a novel study. Interestingly, it was observed that, in cloned and parthenogenetic sheep embryos, there were no differences in the segmentation rate at the evaluated concentrations of OF and UF vs. the control group. This is in agreement with what has been reported for bovine embryos produced using IVF [23,24,25,40]. At the lowest concentration (0.5%), OF and UF showed no effect on the development of the cloned nor parthenogenetic embryos. However, at 1.0%, OF and UF reduced the morula and blastocyst rates in the cloned sheep embryos. An exacerbated effect was observed at 2.0% OF and UF in the parthenogenetic embryos, which were blocked at the 4-to-16-cell stage. In pig-cloned embryos, it was reported that supplementation with 14 and 28 μg mL^−1^ of OF to PZM-5 medium significantly increased the blastocyst rate (27 and 26%, respectively) with respect to the control group (14%). But at higher levels (56 and 100 μg mL^−1^), it had no effect (18.1 and 19.2%) [41]. Unlike the authors, who supplemented OF in μg mL^−1^ in this study, fluids were added as percentage solutions. The differences in the results between both studies could be due to the compounds generated by the metabolism of embryos and the degradation of carbohydrates, amino acids, and proteins in the culture medium, which could accumulate until reaching toxic levels. Therefore, it is essential to maintain an ideal concentration of reproductive fluids that benefit embryonic development without generating high levels of toxic compounds for embryos.

Other studies found that high levels of OF (5, 10, and 25%) or UF (2.5 and 5%) had a negative effect on the blastocyst rate in embryos produced using IVF (11, 10, and 1% for OF; 13.2 and 3% for UF) [24,25]. The data are similar to those observed in this study for cloned embryos at 1% OF and UF, and for parthenogenetic embryos that blocked their development at cleavage at 2.0% OF and UF.

Lopera-Vasquez et al. [24] supplemented SOF medium with 0.625, 1.25, and 2.5% OF for bovine embryos produced using IVF, observing that on day 7, the blastocyst rate was significantly lower at all OF concentrations (16, 17, and 13.9%) when compared to the control group (22.9%). However, on day 9, the blastocyst rate was not different from the control group at 0.625 and 1.25% OF, similar to what occurred in this study for blastocyst rates in cloned embryos treated with 1.0% OF and UF on day 7 of culture.

The most abundant OF proteins during the estrus phase in sheep are oviductin (OVGP1), isocitrate dehydrogenase, elongation factor 1-α1, 71 kDa heat shock protein, є 14-3-3 protein, and annexin A8. Meanwhile, in the luteal phase, the most abundant proteins are α-2 macrobulin, ceruloplasmin, gelsolin, transferrin, and complement factor B [22].

Among these, OVGP1 is synthesized specifically in the oviduct of mammals [42]. This protein plays a predominant role during fertilization, since it regulates polyspermy by hardening the ZP [43]. It also increases the segmentation rate of morulae and blastocysts in goats [42]. Furthermore, the interaction of OVGP1 has been detected by immunohistochemistry in 4–8 cells and morulae bovine embryos, observing to be present in the perivitelline space and within the blastomeres [44]. However, high concentrations of this protein (50 and 100 μg.mL^−1^) have a detrimental effect on the embryonic development in goats [42,45]. Therefore, it is important to supplement OF at an ideal, gradual, and spatial–temporal concentration in in vitro culture systems. In a previous work, we identified the presence of heat shock protein 70 (HSP70) in OF, with an almost null presence of it in UF [46]. However, other studies have identified this protein in UF [47,48], as well as other protein members of this family [43,44]. HSP70 belongs to a family of “chaperone” proteins that perform a wide variety of cellular maintenance activities and counteract the effects caused by thermal stress [49]. It also has an important role in cell function, as the blastocyst rate is reduced when HSP70 function is inhibited during 2-cell bovine embryo culture, even at physiological temperatures (38.5 °C) [50]. The effect that reproductive fluids have on the in vitro development of embryos depends on their characteristics conditioned by variables intrinsic to the origin of the fluids: whether they are collected in vivo or postmortem and the recovery method (by mechanical pressure with forceps, by compression with a slide, by aspiration with an automatic pipette, by scraping with a curette, or by washing with physiological solution) [51,52]. Depending on the recovery method, the fluids may contain a greater or lesser number of impurities such as erythrocytes, oviductal cells, and cellular debris, which are not always efficiently eliminated by centrifugation [51]. Another variable to consider is whether the fluids are pure and concentrated or diluted, which is important since most studies carry out supplementation (v/v) without considering the concentration of its components, which can vary from one to another sample and may not be reproducible. The best would be the supplementation of these fluids based on their protein concentration (μg mL^−1^), which are the main molecules that constitute them and which culture media lack.

Regarding the levels of ROS and GSH present in sheep parthenogenetic embryos, in this study it was observed that, at 1.0% OF and UF, the levels of both decreased significantly in the blastocysts, results similar to those reported in other studies, where ROS concentrations, as well as the expression of genes related to oxidative stress decreased in bovine embryos produced using IVF and treated with OF and UF [23,25]. In the proteome of the sheep reproductive system, 940 proteins have been identified, 4% related to oxidation–reduction processes [22] and 5% to oxidative stress [47]. Some of the most abundant proteins in OF in the luteal phase are ceruloplasmin and lactotransferrin while, in UF, they are peroxiredoxin-1 and glutathione S-transferase [22,47]. These proteins are part of the non-enzymatic antioxidant system of reproductive fluids and are probably responsible for lowering intracellular ROS levels in parthenogenetic embryos, which is why these embryos did not need to the increase intracellular GSH to counteract the ROS. Hence, by adding 1.0% OF and UF, these levels will decrease. The presence of melatonin in OF, as well as its role in counteracting ROS during the in vitro development of rabbit embryos has also been identified [53,54]. Supplementation with 10 nM of melatonin reduces ROS and increases the blastocyst rate in cloned sheep embryos [30].

For cloned embryos, no effects on the levels of ROS and GSH were observed for the different OF and UF treatments. Likewise, the ROS levels were similar between the cloned and parthenogenetic embryos, which is noteworthy, given the intense manipulation to which the former are subjected that causes exposure to high concentrations of oxygen, changes in temperature, and prolonged exposure to light [32,55]. The opposite was observed for GSH, the levels of which were different between both types of embryos, being about 30% more concentrated in the cloned embryos than in the parthenogenetic ones. This was possibly because two cytoplasts were used to form each cloned embryo, which retained around 70% of the initial volume of the cytoplasm after enucleation. Therefore, each cloned embryo had about 40% more cytoplasm than the parthenogenetic embryos, which increased the intracellular concentration of GSH and possibly this kept the ROS levels in balance in the cloned embryos [56].

It has been described that increasing the cytoplasmic volume of cloned embryos improves their quality and implantation potential due to the addition of mitochondria and other cytoplasmic factors that help nuclear reprogramming [57,58,59], but its effect on intracellular GSH levels has not been described.

Glutathione (GSH) is a non-enzymatic antioxidant synthesized mainly in the cytosol (80–85%) and mitochondria (10–15%), and in lesser quantities, in the nucleus and endoplasmic reticulum. Its antioxidant action can be direct or as a cofactor of antioxidant and detoxifying enzymes. It acts on various free radicals and pro-oxidants, such as hydrogen peroxide, which is a precursor of ROS [60].

Among the main amino acids present in OF, are alanine, glutamic acid, lysine, and glycine [61]. In sheep, OF supplies alanine and glycine, which represent 45% of the total amino acids [62]. Cysteine, glycine, and glutamic acids are precursors to the tripeptide GSH. As these amino acids are found in considerable quantities in OF, it can be inferred that this fluid would promote the synthesis of GSH reducing ROS. Yet, in this study, no increase in the intracellular GSH was observed in the embryos treated with OF or UF.

This study is the first to evaluate the effects of OF and UF on the development rate and quality of cloned sheep embryos produced using HMC. Still, due to the characteristics of this technique, where cloned embryos lack ZP and must be cultured in a WOW system, it made the sequential supplementation with OF and UF complicated.

This study elucidates some effects that OF and UF have on the development rate in cloned sheep embryos and provides the possibility for further research on the impact they may have on the quality of cloned embryos, in terms of the total cell number, gene expression, and epigenetic regulation.

## 5. Conclusions

In this study, no effect on the development rate of cloned vs. parthenogenetic *Ovis aries* embryos was observed when supplementing the culture media with 0.5% OF and UF. Moreover, higher levels had negative effects on the first divisions of the in vitro development in the parthenogenetic embryos, and on the blastocyst stage for the cloned embryos. Still, a positive effect on redox regulation was observed at 1% OF in the parthenogenetic embryos but not in the cloned embryos. We conclude that supplementation with OF and UF did not improve the in vitro development nor the quality of cloned *Ovis aries* embryos produced using HMC.

## Figures and Tables

**Table 1:** Effects of OF and UF on the in vitro development of parthenogenetic *O. aries* embryos.

OF–UF (%)	No.	Cleavage	4 to 16 Cells	Morula	Blastocysts	Fragmented
0.0	140	82.9 ± 3.6 ^a^ (116)	34.5 ± 4.9 ^a^ (40)	53.4 ± 8.1 ^a^ (62)	43.1 ± 7.5 ^a^ (50)	12.1 ± 8.6 ^a^ (14)
0.5	132	81.1 ± 4.0 ^a^ (107)	45.8 ± 6.5 ^a^ (49)	48.6 ± 6.7 ^a^ (52)	31.8 ± 5.7 ^a^ (34)	5.6 ± 4.8 ^a^ (6)
1.0	129	73.6 ± 6.1 ^a^ (95)	32.6 ± 10.9 ^a^ (31)	57.9 ± 12.6 ^a^ (55)	29.5 ± 4.0 ^a^ (28)	9.5 ± 3.9 ^a^ (9)
2.0	55	74.5 ± 12.5 ^a^ (41)	100 ^b^ (41)	0 ^b^	0 ^b^	0 ^a^

Means ± SE (%) of six repetitions were carried out for each treatment. The cleavage rate was determined after 36 h of in vitro culture. The rates of parthenogenetic embryos of the 4 to 16 cells, morula stages, and fragmented embryos were determined on day 4 of the culture. The blastocyst rate was determined on day 7. Different letters (a, b) indicate mean statistical differences among the columns (*p* < 0.05).

**Table 2:** Effects of OF and UF on the in vitro development of cloned *O. aries* embryos.

OF–UF (%)	No.	Cleavage	4 to 16 cells	Morula	Blastocysts	Fragmented
0.0	78	97.4 ± 2.9 ^a^ (76)	26.3 ± 8.0 ^a^ (20)	48.7 ± 9.2 ^a^ (37)	27.6 ± 5.5 ^a^ (21)	25.0 ± 3.7 ^a^ (19)
0.5	76	98.7 ± 0.9 ^a^ (75)	37.3 ± 9.4 ^a^ (28)	45.3 ± 6.5 ^a^ (34)	20.0 ± 4.1 ^a^ (15)	17.3 ± 4.7 ^a^ (13)
1.0	66	100 ^a^ (66)	62.1 ± 7.0 ^a^ (41)	27.3 ± 7.0 ^b^ (18)	7.6 ± 3.0 ^b^ (5)	10.6 ± 4.0 ^a^ (7)

Means ± SE (%) of seven repetitions were carried out for each treatment. The cleavage rate was determined after 36 h of in vitro culture. The rates of cloned embryos at the 4 to 16 cells, morula stages, and fragmented embryos were determined at day 4 of the culture. The blastocyst rate was determined on day 7. Different letters (a, b) indicate mean statistical differences among the columns (*p* < 0.05).

## Data Availability

The data are available from the first author, José Roberto Vazquez-Avendaño (robertmizer@gmail.com), upon request.

## References

[1] Viana J.H.M. (2022). Statistics of embryo production and transfer in domestic farm animals. Embryo Technol. Newsl..

[2] Galli C., Lazzari G. (2021). 25th Anniversary of cloning by somatic-cell nuclear transfer: Current applications of SCNT in advanced breeding and genome editing in livestock. Reproduction.

[3] Borges A.A., Pereira A.F. (2019). Potential role of intraspecific and interspecific cloning in the conservation of wild mammals. Zygote.

[4] Bolton R.L., Mooney A., Pettit M.T., Bolton E.A., Morgan L., Drake G.J., Appeltant R., Walker S.L., Gillis J.D., Hvilsom C. (2022). Resurrecting biodiversity: Advanced assisted reproductive technologies and biobanking. Reprod. Fertil..

[5] Loi P., Palazzese L., Scapolo P.A., Fulka J., Fulka H., Czernik M. (2021). 25th Anniversary of Cloning by Somatic-Cell Nuclear Transfer Scientific and technological approaches to improve SCNT efficiency in farm animals and pets. Reproduction.

[6] Kalds P., Gao Y., Zhou S., Cai B., Huang X., Wang X., Chen Y. (2020). Redesigning small ruminant genomes with CRISPR toolkit: Overview and perspectives. Theriogenology.

[7] Vazquez-Avendaño J.R., Ambríz-García D.A., Cortez-Romero C., Trejo-Córdova A., Navarro-Maldonado M.d.C. (2022). Current state of the efficiency of sheep embryo production through somatic cell nuclear transfer. Small Rumin. Res..

[8] Simmet K., Wolf E., Zakhartchenko V. (2020). Manipulating the Epigenome in Nuclear Transfer Cloning: Where, When and How. Int. J. Mol. Sci..

[9] Sawai K., Takahashi M., Fujii T., Moriyasu S., Hirayama H., Minamihashi A., Hashizume T., Onoe S. (2011). DNA Methylation Status of Bovine Blastocyst Embryos Obtained from Various Procedures. J. Reprod. Dev..

[10] Beaujean N., Taylor J., Gardner J., Wilmut I., Meehan R., Young L. (2004). Effect of Limited DNA Methylation Reprogramming in the Normal Sheep Embryo on Somatic Cell Nuclear Transfer1. Biol. Reprod..

[11] Loi P., Clinton M., Vackova I., Fulka J., Feil R., Palmieri C., Della Salda L., Ptak G. (2005). Placental abnormalities associated with post-natal mortality in sheep somatic cell clones. Theriogenology.

[12] Palmieri C., Loi P., Ptak G., Della Salda L. (2008). Review Paper: A Review of the Pathology of Abnormal Placentae of Somatic Cell Nuclear Transfer Clone Pregnancies in Cattle, Sheep, and Mice. Vet. Pathol..

[13] Ni W., You S., Cao Y., Li C., Wei J., Wang D., Qiao J., Zhao X., Hu S., Quan R. (2016). Aberrant expression of miR-127, miR-21 and miR-16 in placentas of deceased cloned sheep. Res. Vet. Sci..

[14] Nava-Trujillo H., Rivera R.M. (2023). Review: Large offspring syndrome in ruminants: Current status and prediction during pregnancy. Animal.

[15] Young L.E., Schnieke A.E., McCreath K.J., Wieckowski S., Konfortova G., Fernandes K., Ptak G., Kind A.J., Wilmut I., Loi P. (2003). Conservation of IGF2-H19 and IGF2R imprinting in sheep: Effects of somatic cell nuclear transfer. Mech. Dev..

[16] Wang F., Pan J., Zhao L.X., Liu Y.Y., Zhang L., Wang S.Y., Li L., Zhou H.M., Zhang D. (2016). Discovery of DNA Methylation Status of Peg3, Cdkn1c and Gtl2 in Cloned and Natural Lambs. Prog. Biochem. Biophys.

[17] Luo J., Zhang Y., Guo Y., Tang H., Wei H., Liu S., Wang X., Wang L., Zhou P. (2017). TRIM28 regulates Igf2-H19 and Dlk1-Gtl2 imprinting by distinct mechanisms during sheep fibroblast proliferation. Gene.

[18] Cajas Y.N., Cañón-Beltrán K., de la Blanca M.G.M., Sánchez J.M., Fernandez-Fuertes B., González E.M., Rizos D. (2021). Role of reproductive fluids and extracellular vesicles in embryo–maternal interaction during early pregnancy in cattle. Reprod. Fertil. Dev..

[19] Coy P., Romar R., Romero-Aguirregomezcorta J. (2022). The embryo culture media in the era of epigenetics: Is it time to go back to nature?. Anim. Reprod..

[20] Calle A., Fernandez-Gonzalez R., Ramos-Ibeas P., Laguna-Barraza R., Perez-Cerezales S., Bermejo-Alvarez P., Ramirez M.A., Gutierrez-Adan A. (2012). Long-term and transgenerational effects of in vitro culture on mouse embryos. Theriogenology.

[21] Milazzotto M.P., Ispada J., de Lima C.B. (2022). Metabolism-epigenetic interactions on in vitro produced embryos. Reprod. Fertil. Dev..

[22] Soleilhavoup C., Riou C., Tsikis G., Labas V., Harichaux G., Kohnke P., Reynaud K., de Graaf S.P., Gerard N., Druart X. (2016). Proteomes of the Female Genital Tract During the Oestrous Cycle. Mol. Cell Proteom..

[23] Cebrian-Serrano A., Salvador I., García-Roselló E., Pericuesta E., Pérez-Cerezales S., Gutierrez-Adán A., Coy P., Silvestre M. (2012). Effect of the Bovine Oviductal Fluid on In Vitro Fertilization, Development and Gene Expression of In Vitro-Produced Bovine Blastocysts. Reprod. Domest. Anim..

[24] Lopera-Vasquez R., Hamdi M., Maillo V., Gutierrez-Adan A., Bermejo-Alvarez P., Ramírez M.Á., Yáñez-Mó M., Rizos D. (2017). Effect of bovine oviductal extracellular vesicles on embryo development and quality in vitro. Reproduction.

[25] Hamdi M., Lopera-Vasquez R., Maillo V., Sanchez-Calabuig M.J., Núnez C., Gutierrez-Adan A., Rizos D. (2018). Bovine oviductal and uterine fluid support in vitro embryo development. Reprod. Fertil. Dev..

[26] Barrera A.D., García E.V., Hamdi M., Sánchez-Calabuig M.J., López-Cardona P., Balvís N.F., Rizos D., Gutiérrez-Adán A. (2017). Embryo culture in presence of oviductal fluid induces DNA methylation changes in bovine blastocysts. Reproduction.

[27] Canovas S., Ivanova E., Romar R., García-Martínez S., Soriano-Úbeda C., García-Vázquez A.F., Saadeh H., Andrews S., Kelsey G., Coy P. (2017). DNA methylation and gene expression changes derived from assisted reproductive technologies can be decreased by reproductive fluids. eLife.

[28] You J., Kim J., Lim J., Lee E. (2010). Anthocyanin stimulates in vitro development of cloned pig embryos by increasing the intracellular glutathione level and inhibiting reactive oxygen species. Theriogenology.

[29] Su J., Wang Y., Xing X., Zhang L., Sun H., Zhang Y. (2015). Melatonin significantly improves the developmental competence of bovine somatic cell nuclear transfer embryos. J. Pineal Res..

[30] Nadri P., Ansari-Mahyari S., Jafarpour F., Mahdavi A.H., Vash N.T., Lachinani L., Dormiani K., Nasr-Esfahani M.H. (2022). Melatonin accelerates the developmental competence and telomere elongation in ovine SCNT embryos. PLoS ONE.

[31] Koo O.J., Jang G., Kwon D.K., Kang J.T., Kwon O.S., Park H.J., Kang S.K., Lee B.C. (2008). Electrical activation induces reactive oxygen species in porcine embryos. Theriogenology.

[32] Deluao J.C., Winstanley Y., Robker R.L., Pacella-Ince L., Gonzalez M.B., McPherson O.N. (2022). Oxidative stress and reproductive function: Reactive oxygen species in the mammalian pre-implantation embryo. Reproduction.

[33] Carrasco L.C., Coy P., Avilés M., Gadea J., Romar R. (2008). Glycosidase determination in bovine oviducal fluid at the follicular and luteal phases of the oestrous cycle. Reprod. Fertil. Dev..

[34] Navarro-Maldonado M.D.C., Hernández-Martínez S., Vázquez-Avendaño J.R., Martínez-Ibarra J.L., Zavala-Vega N.L., Vargas-Miranda B., Rivera-Rebolledo J.A., Ambríz-García D.A. (2015). Deriva de células epiteliales de tejido de piel descongelado de Ovis canadensis mexicana para la formación de un banco de germoplasma. Acta Zoo. Mex..

[35] Ward F., Lonergan P., Enright B., Boland M. (2000). Factors affecting recovery and quality of oocytes for bovine embryo production in vitro using ovum pick-up technology. Theriogenology.

[36] Vazquez-Avendaño J.R., Hernández-Martínez S., Hernández-Pichardo J.E., Rivera-Rebolledo J.A., Ambriz-García D.A., Navarro-Maldonado M.D.C. (2017). Efecto del uso de medio secuencial humano en la producción de blastocistos de hembra ovis canadensis mexicana por clonación manual. Acta Zoo. Mex..

[37] Martínez S.H., Pichardo J.E.H., Avendaño J.R.V., García D.A.A., Maldonado M.D.C.N. (2020). Developmental dynamics of cloned Mexican bighorn sheep embryos using morphological quality standards. Vet. Med. Sci..

[38] Vajta G., Lewis I.M., Hyttel P., Thouas G.A., Trounson A.O. (2001). Somatic Cell Cloning without Micromanipulators. Cloning.

[39] Vajta G., Korösi T., Du Y., Nakata K., Ieda S., Kuwayama M., Nagy Z.P. (2008). The Well-of-the-Well system: An efficient approach to improve embryo development. Reprod. Biomed. Online.

[40] Nina M., Ayala C., Susaño R. (2021). Fluido uterino de llama (Lama glama), como medio para potenciar el desarrollo embrionario de vacas (Bos taurus) en cultivos in vitro. RIIARn.

[41] Zhang Y.H., Song E.S., Kim E.S., Cong P.Q., Lee S.H., Lee J.W., Yi Y.J., Park C.S. (2009). Effects of Oviductal Fluid, Culture Media and Zona Pellucida Removal on the Development of Porcine Embryos by Nuclear Transfer. Asian Australas. J. Anim. Sci..

[42] Pradeep M., Jagadeesh J., De A., Kaushik J., Malakar D., Kumar S., Dang A., Das S., Mohanty A. (2011). Purification, sequence characterization and effect of goat oviduct-specific glycoprotein on in vitro embryo development. Theriogenology.

[43] Bragança G., Alcântara-Neto A., Batista R., Brandão F., Freitas V., Mermillod P., Souza-Fabjan J. (2021). Oviduct fluid during IVF moderately modulates polyspermy in in vitro-produced goat embryos during the non-breeding season. Theriogenology.

[44] Banliat C., Tsikis G., Labas V., Teixeira-Gomes A.-P., Com E., Lavigne R., Pineau C., Guyonnet B., Mermillod P., Saint-Dizier M. (2020). Identification of 56 Proteins Involved in Embryo–Maternal Interactions in the Bovine Oviduct. Int. J. Mol. Sci..

[45] Algarra B., Maillo V., Avilés M., Gutiérrez-Adán A., Rizos D., Jiménez-Movilla M. (2018). Effects of recombinant OVGP1 protein on in vitro bovine embryo development. J. Reprod. Dev..

[46] Avendaño J.R.V., Romero C.C., García D.A.A., Barrera M.D.L.A.F., Ortega M.P.C., Rodríguez H.L., Flores G.B., Maldonado M.d.C.N. (2024). Physicochemical characteristics and protein profile of oviductal and uterine fluids from domestic sheep. Austral. J. Vet. Sci..

[47] Koch J.M., Ramadoss J., Magness R.R. (2010). Proteomic Profile of Uterine Luminal Fluid from Early Pregnant Ewes. J. Proteome Res..

[48] Burns G., Brooks K., Wildung M., Navakanitworakul R., Christenson L.K., Spencer T.E. (2014). Extracellular Vesicles in Luminal Fluid of the Ovine Uterus. PLoS ONE.

[49] Rosenzweig R., Nillegoda N.B., Mayer M.P., Bukau B. (2019). The Hsp70 chaperone network. Nat. Rev. Mol. Cell Biol..

[50] Al-Katanani Y., Hansen P. (2002). Induced thermotolerance in bovine two-cell embryos and the role of heat shock protein 70 in embryonic development. Mol. Reprod. Dev..

[51] Velazquez M., Parrilla I., Van Soom A., Verberckmoes S., Kues W., Niemann H. (2010). Sampling techniques for oviductal and uterine luminal fluid in cattle. Theriogenology.

[52] Itze-Mayrhofer C., Brem G. (2020). Quantitative proteomic strategies to study reproduction in farm animals: Female reproductive fluids. J. Proteom..

[53] Voiculescu S., Zygouropoulos N., Zahiu C., Zagrean A. (2014). Role of melatonin in embryo fetal development. J. Med. Life.

[54] Qu P., Luo S., Du Y., Zhang Y., Song X., Yuan X., Lin Z., Li Y., Liu E. (2020). Extracellular vesicles and melatonin benefit embryonic develop by regulating reactive oxygen species and 5-methylcytosine. J. Pineal Res..

[55] Soto-Heras S., Paramio M.-T. (2020). Impact of oxidative stress on oocyte competence for in vitro embryo production programs. Res. Vet. Sci..

[56] Liu R.-H., Li Y.-H., Jiao L.-H., Wang X.-N., Wang H., Wang W.-H. (2002). Extracellular and intracellular factors affecting nuclear and cytoplasmic maturation of porcine oocytes collected from different sizes of follicles. Zygote.

[57] Panda S.K., George A., Saha A.P., Sharma R., Manik R.S., Chauhan M.S., Palta P., Singla S.K. (2011). Effect of Cytoplasmic Volume on Developmental Competence of Buffalo (*Bubalus bubalis*) Embryos Produced Through Hand-Made Cloning. Cell Reprogramming.

[58] Liu X., Luo C., Deng K., Wu Z., Wei Y., Jiang J., Lu F., Shi D., Liu X., Luo C. (2018). Cytoplasmic volume of recipient oocytes affects the nucleus reprogramming and the developmental competence of HMC buffalo embryos. J. Vet. Med. Sci..

[59] Raja A., Sahare A., Jyotsana B., Priya D., Palta P., Chauhan M., Manik R., Singla S. (2019). Reducing the cytoplasmic volume during hand-made cloning adversely affects the developmental competence and quality, and alters relative abundance of mRNA transcripts and epigenetic status of buffalo (Bubalus bubalis) embryos. Anim. Reprod. Sci..

[60] Averill-Bates D.A. (2023). The antioxidant glutathione. Vitam. Horm..

[61] Saint-Dizier M., Schoen J., Chen S., Banliat C., Mermillod P. (2019). Composing the Early Embryonic Microenvironment: Physiology and Regulation of Oviductal Secretions. Int. J. Mol. Sci..

[62] Wales R. (1973). The Uterus of the Ewe II. Chemical Analysis of Uterine Fluid Collected by Cannulation. Aust. J. Biol. Sci..

